# Clustering of Cardiovascular Risk Factors and Heart Failure in Older Adults from the Brazilian Far North

**DOI:** 10.3390/healthcare12090951

**Published:** 2024-05-06

**Authors:** Guilherme José Silva Ribeiro, Emilio Hideyuki Moriguchi, André Araújo Pinto

**Affiliations:** 1 Graduate Program in Nutrition Science, Department of Nutrition, Federal University of Viçosa, Viçosa 36570-900, Brazil; guilherme.j.ribeiro@ufv.br; 2 Graduate Program in Cardiology and Cardiovascular Sciences, Department of Cardiology, Federal University of Rio Grande do Sul, Rio Grande do Sul 90010-150, Brazil; emoriguchi@hcpa.edu.br; 3 Health Sciences Center, State University of Roraima, Roraima 69306-530, Brazil

**Keywords:** heart failure, aged, risk factors, clustering, primary health care

## Abstract

Given the aging global population, identifying heart failure (HF) phenotypes has become crucial, as distinct disease characteristics can influence treatment and prognosis in older adults. This study aimed to analyze the association between clustering of cardiovascular risk factors and HF in older adults. A cross-sectional epidemiological study was conducted with 1322 older adults (55% women, mean age 70.4) seen in primary health care. Diagnosis of HF was performed by a cardiologist based on diagnostic tests and medical history. Cardiovascular risk factors included hypertension, diabetes, hypercholesterolemia, and smoking. Using logistic regression, potential associations were tested. Individual risk factor analysis showed that older adults with hypertension, diabetes, or hypercholesterolemia had up to 7.6 times higher odds to have HF. The cluster where older adults had only one risk factor instead of none increased the odds of HF by 53.0%. Additionally, the odds of older patients having HF ranged from 3.59 times for the two-risk factor cluster to 20.61 times for the simultaneous presence of all four factors. The analysis of clusters substantially increasing HF risk in older adults revealed the importance of individualizing subgroups with distinct HF pathophysiologies. The clinical significance of these clusters can be beneficial in guiding a more personalized therapeutic approach.

## 1. Introduction

As the global population ages, the high prevalence of heart failure (HF) among older adults with multiple comorbidities places significant strain on global healthcare systems [1,2]. HF is a clinical syndrome characterized by symptoms such as shortness of breath and ankle swelling, resulting from structural or functional abnormalities of the heart, leading to elevated pressures and inadequate cardiac output [3]. Unfortunately, older adult HF patients tend to experience high rates of mortality (19.2%), hospitalization (16.7%), or both events (29.8%) within a short two-year period [4]. Thus, neglecting HF in older adults implies subjecting them to serious consequences, both for individual health and support networks.

HF is associated with several comorbidities that negatively impact clinical outcomes in older adults, often due to neurohormonal overload, inflammatory activation [5], oxidative stress, and endothelial dysfunction [6]. In this context, cluster analyses have been recommended to identify groups with specific combinations of comorbidities, suggested as representative of patients with common pathophysiological mechanisms [7,8,9]. Among the comorbidities related to HF in older adults, traditional cardiovascular risk factors, including hypertension [7,8,9], alcohol consumption [9], diabetes [10,11], hypercholesterolemia [12,13,14], and smoking [15], play a fundamental role.

Cluster studies on the topic of HF typically explore the relationship between the presence of multiple cardiovascular risk factors and the development or worsening of HF [16,17,18,19,20,21]. These studies tend to show that the simultaneous presence of various cardiovascular risk factors significantly increases the risk of developing HF. Additionally, they underscore that clustering these risk factors may contribute to disease progression and worsen clinical outcomes for HF patients [16,17,18,19,20,21].

These findings raise concerns for Brazil, which experienced a significant increase of 57.4% in HF incidence in its older adult population between 2010 and 2022 [22]. However, despite demographic growth, understanding of HF in Brazil remains incomplete, as revealed by a recent systematic review [23]. Among the 13 studies analyzed, heterogeneities were observed, including sample sizes ranging from 25 to 2056 participants, as well as different types of designs and treatment locations, both outpatient and inpatient. Surprisingly, some of the prevalences found exceeded 50%, and none of the available studies analyzed the association between HF and clustering of cardiovascular risk factors [23].

The Brazilian scenario becomes more concerning when examining the landscape of cardiovascular risk factors, based on the Surveillance System for Risk and Protection Factors for Chronic Diseases by Telephone Survey conducted in 2023 [24]. Although smoking (9.7%), hypertension (65.1%), diabetes (30.6%) [24], and hypercholesterolemia [25] prevalences in the older adult population reveal a favorable scenario for HF, the influence of the clustering of these factors in amplifying the risk of development and worsening of the disease remains uncertain. It is postulated that the clustering of cardiovascular risk factors, including hypertension, diabetes, hypercholesterolemia, and smoking, is significantly associated with HF occurrence in Brazilian older adults.

Even though there have been advances in the treatment of HF, many patients still face residual risks of adverse events, necessitating the exploration of new therapeutic targets [26]. Given HF’s propensity to predominantly affect older adult patients and the scarcity of data on its clinical characteristics in Brazilian populations [23], generating epidemiological information is crucial. These data are essential for guiding healthcare professionals and policymakers in managing HF, identifying high-risk groups, and potentially improving patient outcomes. Furthermore, understanding how cardiovascular risk factors cluster can inform HF prevention and management strategies. Therefore, this study investigates the association between clustered cardiovascular risk factors and HF in older adults.

## 2. Materials and Methods

### 2.1. Study Design and Location

This study was conducted in the state of Roraima, Brazil, in Primary health care. Related data fall under the jurisdiction of the Epidemiological Surveillance Department (ESD) of the Roraima State Department of Health. This study is a cross-sectional observational epidemiological study that is linked to a larger study focused on the health conditions of older adults in the state. Data were collected from January to December 2020 by health professionals working in primary health care. The study was ethically approved by the State University of Roraima’s Research Ethics Committee (protocol no. 5385012), adhering to the Declaration of Helsinki and National Health Council guidelines.

Roraima, the least populous state in Brazil, is home to 636,707 inhabitants and covers an area of 223,644 km^2^, resulting in a population density of 2.85 inhabitants/km^2^. Located in the far north of the country, Roraima has only 15 municipalities, with the majority concentrated around the capital, Boa Vista, where 65% of the state population resides. In 2022, the average monthly income per person in households reached R$1242 (approximately 287 dollars), placing the state 15th among the 27 Brazilian states in terms of income. Roraima’s Human Development Index (HDI) is 0.707, reflecting progress in education, income, and an estimated life expectancy of 71.8 years. These characteristics provide the context for investigating the association of cardiovascular risk factors with HF.

### 2.2. Population and Sample

According to the ESD, there were 4194 medical records available for consultation. However, during data screening, many incomplete medical records were observed, prompting the need for a minimum sample estimate, which would enable the exclusion of incomplete medical records, eliminating the need for weighting procedures. For this, an estimated prevalence of 50% (for unknown outcomes), a confidence interval of 95%, a tolerable error of 4 percentage points, and a design effect (deff) set at 2 (multiplier factor) were considered. Additionally, 20% was added to mitigate losses. Based on these parameters, a minimum of 1260 complete records was necessary. Despite the many incomplete pieces of information, partly attributed to not all data being entered into the ESD system, the final sample remained robust, totaling 1322 older adults.

**Figure 1:**
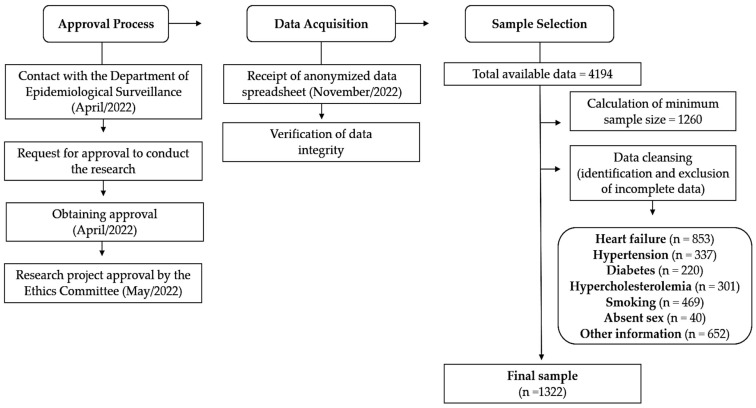
Flowchart of the process of obtaining the final sample included in the present study.

Using a standard form widely employed in Brazilian basic health units, health professionals recorded sociodemographic and health information of older adults, which was later inputted into the ESD system in a binary format (yes or no). The state health authorities provided the data in November 2022 through an electronic spreadsheet obtained from the ESD control system, following strict confidentiality protocols and with written consent obtained. All older adults (individuals aged 60 years or older) residing in the state of Roraima and receiving care in primary health care were considered eligible. While the history of cardiovascular events in the target population was taken into account during medical consultations, older adults were considered eligible regardless of the presence or absence of these outcomes. Incomplete data from older adult patients lacking information (yes or no) on HF, hypertension, diabetes, hypercholesterolemia, smoking status, or sociodemographic details necessary for the identification of older adults were excluded. The final sample was established as shown in Figure 1.

### 2.3. Outcome

The diagnostic conclusion of HF was conducted by primary health care cardiologists, adhering to the Brazilian Heart Failure Guidelines [27]. Initially, HF suspicion, categorized as low, intermediate, or high, was established based on patient medical history analysis. Subsequently, brain natriuretic peptide (BNP) levels were measured, with values above 25–50 pg/mL confirming intermediate to high suspicion. Regardless of suspicion level, all patients underwent an echocardiogram to assess cardiac structure, function, and prognostic stratification. Left ventricular ejection fraction (LVEF) was evaluated during the echocardiogram. For patients whose initial assessment left them in a gray area regarding the diagnosis of HF (for example, with intermediate suspicion or borderline BNP levels), additional diagnostic tests were utilized (e.g., hemodynamic stress tests). The physician then diagnosed HF according to the following classifications: HF with reduced ejection fraction (LVEF < 40%), HF with intermediate ejection fraction (LVEF between 40% and 50%), and HF with preserved ejection fraction (LVEF > 50%). For patients without HF, criteria included (i) low suspicion based on medical history analysis and (ii) BNP levels below 25–50 pg/mL. However, only information about the presence or absence of HF was entered into the Department of Surveillance control system, without distinction for the type of ejection fraction. Therefore, all older adult individuals were included in the analysis regardless of the type of ejection fraction.

### 2.4. Exposure

Traditional cardiovascular risk factors were diagnosed by specialized physicians following recommendations from the Brazilian Ministry of Health. The criteria and diagnostic definitions followed recommendations from the Brazilian Society of Cardiology [28], the Brazilian Society of Diabetes Mellitus [29], and the Brazilian Guideline for Dyslipidemia and Atherosclerosis Prevention [30]. Arterial hypertension was diagnosed according to the maintenance of blood pressure values equal to or greater than 140/90 mmHg [28]. Diabetes diagnosis was based on a fasting plasma glucose level equal to or greater than 126 mg/dL (7.0 mmol/L) after an overnight fast, or hemoglobin A1c equal to or greater than 6.5% [29]. Hypercholesterolemia was defined as the presence of serum levels of low-density lipoprotein (LDL) equal to or greater than 130 mg/dL [30], confirmed through laboratory diagnosis. Following recommendations from the Brazilian Ministry of Health, via the National Cancer Institute, smoking was defined as regular and continuous consumption of tobacco products, such as cigarettes, cigars, or pipes. Smoking status was assessed with the question “Do you smoke?”, with response options “yes” or “no”.

### 2.5. Covariates

Recorded covariates were sex (male or female) and age (recorded in completed years), also categorized into age groups. Additionally, skin color or race was classified according to the national classification system, where individuals self-declared their skin color or race from the available categories: yellow, white, black, brown, and indigenous. Information was also collected on the place of residence (capital or interior) and the education level of older adults, which, due to diverse responses, was categorized into “no education”, “<8 years”, or “≥8 years”.

### 2.6. Statistical Analysis

The general characteristics of the participants were described through frequency distributions, both absolute and relative. Each cardiovascular risk factor was considered as an independent variable and examined both individually and in clusters. In cluster analysis, two variables were created. The first related to the clustering of the four independent variables (hypertension, diabetes, hypercholesterolemia, and smoking), forming 16 possible combinations. The second considered the number of cardiovascular risk factors at the individual level (zero, one, two, three, or four risk factors). To calculate the expected prevalence (E), the individual probability of each risk factor was multiplied by its observed prevalence (O). When a risk factor was absent among the clusters, we multiplied by the inverse of its observed prevalence (1–O). Thus, if the ratio of observed to expected prevalence (O/E) was greater than 1, it indicated the presence of clustering [31].

Logistic regression analyses (both crude and adjusted) were used to verify associations between clustering of cardiovascular risk factors and HF. In the multivariate analysis, two models (both adjusted for all covariates) were executed, with the first considering individual risk factors, and the second considering clustering. All data were analyzed using IBM^®^ SPSS^®^ software (version 26), with a significance level of 5%.

## 3. Results

The general characteristics of the 1322 study patients are presented in Table 1. The average age of the older adults was 70.4 (SD = 7.87) years. They were predominantly female, aged between 60 and 69 years, of mixed race, lacked formal education, and resided in the capital. The prevalences of hypertension, diabetes, hypercholesterolemia, and smoking were 75.3%, 51.9%, 54.4%, and 11.6%, respectively. For all cardiovascular risk factors, an interaction was observed with sociodemographic variables (*p* < 0.05), except between education and hypercholesterolemia (*p* = 0.106) and education and smoking (*p* = 0.144).

Table 2 presents the results of the prevalence of observed and expected clustering of cardiovascular risk factors among the older adult study participants. The observed prevalence of clustering of the four cardiovascular risk factors at the individual level was 4.1%, which was 33.3% higher (O/E = 1.1) than expected for a random event. The most frequent simultaneous cardiovascular risk factors for one, two, and three risk factors were hypertension (54.0%), diabetes and hypercholesterolemia (37.0%), and hypertension, diabetes, and hypercholesterolemia (35.0%), respectively. Finally, the prevalence of non-clustering of cardiovascular risk factors was 4.33 times higher than the expected prevalence.

The individual prevalences of hypertension, diabetes, hypercholesterolemia, and smoking among older adults with CHF were 21.0%, 27.8%, 25.5%, and 17.2%, respectively. In the crude analysis of individual cardiovascular risk factors, only smoking did not show a significant association with the outcome (*p* = 0.614). When grouping the risk factors, the significant associations observed in the crude analysis remained significant in the adjusted analysis. Overall, a direct trend was observed between the number of clustered factors and the risk of HF. Older adults who had one or more risk factors were up to 20 times more likely (OR = 20.61; 95% CI = 5.62–36.62) to have HF compared to their counterparts with no risk factors. The crude and adjusted ORs for individual and clustered cardiovascular risk factors can be found in Table 3.

## 4. Discussion

The results of this study underscore the relationship between cardiovascular risk factors and HF in older adults. The analysis indicates that most individual cardiovascular risk factors, except for smoking, were associated with HF in this population. Notably, it was found that the clustering of two or more risk factors was present in three-quarters of the older adults studied, indicating a high prevalence of multiple risk conditions. Furthermore, the findings suggest that the presence of a greater number of risk factors is associated with a substantial increase in the risk of HF, demonstrating a dose-response relationship. These results are of great clinical and epidemiological importance as they provide a better understanding of the management of HF in older adults. Early identification and effective control of cardiovascular risk factors, including pressure overload, may represent a decisive strategy in the prevention and treatment of this debilitating condition, with potential significant benefits for the health and well-being of this vulnerable population.

The results revealed that the clustering of cardiovascular risk factors significantly increases the likelihood of HF in older adults. Although this investigation was limited to four risk factors, its findings corroborate previous studies that have explored the clustering of two [16,17] and three or more risk factors for HF [18,20]. This finding translates into clinical challenges in managing these patients, as the lack of effective therapies for HF, partially explained by patient heterogeneity, contributes to a mortality rate of 50% over 5 years [21]. Indeed, HF is inherently heterogeneous by definition, as it represents the outcome of a wide range of cardiovascular diseases and risk factors [32]. We hypothesize that the clustering of cardiovascular risk factors chronically overloads the heart, resulting in progressive cardiac remodeling, systolic and/or diastolic dysfunction, and eventually HF. In this sense, therapeutic personalization across the spectrum of HF is imperative, as the numerous patient subgroups with the disease may not benefit from a single therapy [33]. By understanding the impact of clustered cardiovascular risk factors on HF development, healthcare professionals and policymakers can implement targeted interventions and preventive strategies to reduce the burden of HF and improve patient outcomes.

Individual risk factors play a crucial role in predisposing older adults to HF, with hypertension increasing the likelihood of the disease by over five times. Previous studies conducted in the United States [7], United Kingdom [8], and Europe [9] have consistently demonstrated that hypertension significantly increases the risk of HF, which is in line with our findings. Furthermore, a previous study has shown that joint risk factor control, including hypertension management, was associated with a substantial reduction in incident HF risk [34]. Traditionally, hypertension was believed to lead to hypertrophied concentration of the left ventricle [35]. However, it has previously been reported that eccentric hypertrophy is also common in hypertensive patients [36]. Another possible explanatory mechanism may be related to arterial media hypertrophy and its implication in cardiac overload. For example, hypertension induces an increase in the media thickness of arteries, especially arterioles, resulting in increased peripheral vascular resistance, potentially amplifying the hemodynamic burden on the myocardium [37]. These findings highlight the importance of strict blood pressure control in older adults to prevent HF. By elucidating the pathways through which hypertension contributes to HF development, healthcare providers can develop more effective strategies for blood pressure management and cardiovascular risk reduction in older adults.

Diabetes is a significant individual cardiovascular risk factor for HF and ranks highest among all investigated factors. This result is consistent with a study involving over 200,000 Americans, which showed that patients with diabetes had an increased risk of HF hospitalization in the following six months [10]. A meta-analysis involving over 12 million individuals from various countries revealed that patients with diabetes face up to a fivefold increase in the likelihood of HF [11]. Furthermore, a meta-analysis of 77 studies found that diabetes is associated with a twofold increased risk of HF in the general population and a 69% increase in patient populations [38]. One explanation for diabetes’s potential to influence HF may be related to the sharing of common risk factors, including reflecting high hospitalization rates [10]. Additionally, diabetes may contribute to the occurrence of diastolic dysfunction or diabetic cardiomyopathy with systolic ventricular dysfunction, thereby independently increasing the risk of HF from other cardiovascular risk factors [39]. In light of this, more assertive preventive strategies related to diabetes may be imperative, aiming to anticipate or delay the onset of hospitalization-prone HF [10]. For example, healthcare providers can implement targeted interventions to mitigate the impact of diabetes on HF outcomes and improve patient care.

The increased risk of HF associated with hypercholesterolemia was also identified in other studies conducted in older adults from Hong Kong [12], the United States [13], and Spain [14]. It is believed that oxidative stress and inflammation triggered by hypercholesterolemia contribute to cardiac remodeling, through processes that include the excessive production of reactive oxygen species, which can cause mitochondrial and cellular damage, consequently favoring HF [40]. Another hypothesis may involve the formation of atherosclerotic plaques and the associated inflammatory response in the cardiovascular system, contributing to the development of atherosclerosis and inflammation in arteries, and increasing the risk of plaque rupture and cardiovascular events, including HF [13]. Particularly, we also believe that hypercholesterolemia may contribute to the development of diabetes and hypertension, both recognized as contributing factors to HF progression, as described earlier. This interconnection between diabetes and hypertension may amplify HF development and progression and underscores the importance of addressing multiple risk factors in the prevention and management of cardiovascular disease.

The main limitations of the study were identified. Firstly, due to the cross-sectional design, it was not possible to establish causal inferences between the outcomes. This limitation suggests a need for future longitudinal research to better understand the temporal sequence of events and causal pathways. Longitudinal studies would allow for the examination of changes over time and the identification of potential predictors or risk factors for HF development. Secondly, the lack of complete outcome data raises the possibility of underreporting, affecting the accuracy and comprehensiveness of the conclusions. Despite the considerable percentage of older adults with missing data, it is unlikely to have introduced bias in the results, given the sample size, which exceeded some previous studies on the topic [23]. However, underreporting may have occurred in the final sample due to general practitioners’ difficulties in diagnosing HF. Many of these physicians were not familiar with the disease progression, and lacked complete knowledge of relevant evidence and guidelines. Thirdly, although data on tobacco consumption were collected by the medical team, the reliance on self-reporting of this factor may have introduced inaccuracies related to participants’ desire to provide socially desirable responses, given the sensitivity of the topic for older adults. Fourthly, the use of dichotomized data, as provided for the present study, contributes to a loss of information about the severity or intensity of the medical condition and statistical sensitivity. Fifthly, the data are limited to older adults from a specific region in Brazil, a country of continental dimensions, where different regions exhibit distinct climatic, cultural, and behavioral characteristics [41]. Consequently, these findings may not be applicable to older adults in other regions. Finally, the absence of information on confounding factors such as lifestyle (e.g., alcohol consumption), income, and others, may influence the relationship between HF and cardiovascular risk factors, constituting a significant limitation.

Among the strengths of the study, the large sample of over 1300 older adults stands out, as well as the attainment of outcomes through nosological diagnosis based on clinical and laboratory tests. The study also benefits from external validity, allowing the generalization of results to other older adults with similar characteristics in Brazil. Finally, the use of cluster analysis, a promising technique that advances our understanding of the clustering of cardiovascular risk factors and HF in older adults, represented a strength of this study.

## 5. Conclusions

The findings of this study underscore the importance of identifying and analyzing clusters of cardiovascular risk factors in understanding HF in older adults. The results indicate that individually, diabetes, hypertension, and hypercholesterolemia, but not smoking, are significantly associated with HF. Additionally, when all these factors are present, including smoking, the odds of HF increase by up to 20 times. Notably, there was a gradual increase in the risk of HF, highlighting the need for a personalized therapeutic approach for older adults with distinct pathophysiologies. The identified clusters can help guide prevention and treatment strategies for managing HF in older adults, significantly improving the quality of life and prognosis of these patients.

## Figures and Tables

**Table 1:** General characteristics of the older adult study participants and distribution of cardiovascular risk factors across different sociodemographic strata (*n* = 1322). Roraima, Brazil, 2020.

Variables	Total	Hypertension	Diabetes	Hypercholesterolemia	Smoking
Sex, *n* (%)					
Male	595 (45.0)	421 (70.8)	283 (47.6)	269 (45.2)	85 (14.3)
Female	727 (55.0)	574 (79.0)	403 (55.4)	450 (61.9)	69 (9.5)
Age group, *n* (%)					
60–69 years	712 (53.9)	480 (67.4)	295 (41.4)	338 (47.7)	98 (13.8)
70–79 years	416 (31.5)	340 (81.7)	265 (63.7)	258 (62.0)	47 (11.3)
≥80 years	194 (14.7)	175 (90.2)	126 (64.9)	123 (63.4)	10 (4.6)
Skin color/race, *n* (%)					
Yellow	61 (4.6)	54 (88.5)	44 (72.1)	43 (70.5)	13 (21.3)
White	302 (22.8)	216 (71.5)	152 (50.3)	172 (57.0)	30 (9.9)
Black	222 (16.8)	175 (78.8)	125 (56.3)	126 (56.8)	22 (9.9)
Brown	597 (45.2)	434 (72.7)	280 (46.9)	291 (48.7)	58 (9.7)
Indigenous	140 (10.6)	116 (82.9)	85 (60.7)	87 (62.1)	31 (22.1)
Education, *n* (%)					
No education	624 (47.2)	510 (81.7)	369 (58.2)	355 (56.9)	81 (13.0)
<8 years	395 (29.9)	286 (72.4)	188 (47.6)	198 (50.1)	47 (11.9)
≥8 years	303 (22.9)	199 (65.7)	135 (44.6)	166 (54.8)	26 (8.6)
Local, *n* (%)					
Capital	847 (64.1)	622 (73.4)	395 (46.6)	428 (50.5)	87 (14.1)
Interior	475 (35.9)	373 (78.5)	291 (61.3)	291 (61.3)	67 (10.3)

Note: n: absolute frequency; (%): relative frequency.

**Table 2:** Clustering of cardiovascular risk factors in older adult study participants. Roraima, Brazil, 2020.

No. Factors	Hypertension	Diabetes	Hypercholesterolemia	Smoking	O (%)	E (%)	O/E (95% CI)
0	-	-	-	-	0.13	0.03	4.3 (3.3–5.2)
1	+	-	-	-	0.54	0.27	2.0 (1.3–2.7)
1	-	+	-	-	0.22	0.07	3.3 (2.4–4.1)
1	-	-	+	-	0.28	0.09	3.0 (2.1–3.8)
1	-	-	-	+	0.09	0.02	3.8 (2.8–4.7)
2	+	+	-	-	0.46	0.19	2.5 (1.7–3.3)
2	+	-	+	-	0.47	0.20	2.4 (1.6–3.1)
2	+	-	-	+	0.09	0.02	4.6 (3.5–5.5)
2	-	+	+	-	0.37	0.04	8.9 (7.3–10.2)
2	-	+	-	+	0.06	0.01	8.9 (7.4–10.2)
2	-	-	+	+	0.06	0.01	8.4 (6.9–9.7)
3	+	+	+	-	0.35	0.31	1.1 (0.6–1.5)
3	+	+	-	+	0.06	0.03	2.2 (1.4–2.9)
3	-	+	+	+	0.04	0.01	4.0 (3.0–4.9)
3	+	-	+	+	0.06	0.03	2.1 (1.4–2.7)
4	+	+	+	+	0.04	0.03	1.1 (0.6–1.5)

Note: No.: number of factors; 0: no factors; 1: only one factor; 2: only two factors clustered; 3: three factors clustered; 4: all factors clustered; O: observed prevalence; E: expected prevalence; O/E: observed/expected ratio; 95% CI: 95% confidence interval; (+): presence of risk factor; (-): absence of risk factor.

**Table 3:** Crude and adjusted OR and 95% CI for the association of cardiovascular risk factors and their clusters with heart failure in older adults. Roraima, Brazil, 2020.

Risk Factors	Heart Failure (%)	Crude	Adjusted *
		OR (95% CI)	OR (95% CI)
Hypertension			
No	4.9	Ref.	Ref.
Yes	21.0	5.16 (3.05–7.28)	5.52 (2.75–8.50)
Diabetes			
No	5.3	Ref.	Ref.
Yes	27.8	6.83 (4.65–7.05)	7.26 (4.56–10.05)
Hypercholesterolemia			
No	7.0	Ref.	Ref.
Yes	25.5	4.56 (3.20–5.97)	4.33 (2.87–6.52)
Smoking			
No	15.6	Ref.	Ref.
Yes	17.2	1.10 (0.56–1.41)	1.16 (0.69–1.64)
Clusters			
No	6.2	Ref.	Ref.
One	7.5	1.21 (1.06–1.64)	1.53 (1.02–2.15)
Two	10.7	1.73 (1.53–1.99)	3.59 (1.50–5.17)
Three	32.2	5.20 (4.59–5.92)	14.20 (4.36–25.29)
Four	32.5	6.24 (4.47–8.86)	20.61 (5.62–36.62)

Note: (%): prevalence of heart failure; OR: odds ratio; 95% CI: 95% confidence interval; * adjusted for sex, continuous age, skin color/race, education, and place of residence.

## Data Availability

All data used and/or analyzed during the current study are available from the corresponding authors upon reasonable request.

## References

[1] Liu E., Lampert B.C. (2022). Heart Failure in Older Adults: Medical Management and Advanced Therapies. Geriatrics.

[2] Okoye C., Mazzarone T., Niccolai F., Bencivenga L., Pescatore G., Bianco M.G., Guerrini C., Giusti A., Guarino D., Virdis A. (2023). Predicting mortality and re-hospitalization for heart failure: A machine-learning and cluster analysis on frailty and comorbidity. Aging. Clin. Exp. Res..

[3] McDonagh T.A., Metra M., Adamo M., Gardner R.S., Baumbach A., Bohm M., Burri H., Butler J., Celutkiene J., Chioncel O. (2021). 2021 ESC Guidelines for the diagnosis and treatment of acute and chronic heart failure: Developed by the Task Force for the diagnosis and treatment of acute and chronic heart failure of the European Society of Cardiology (ESC) With the special contribution of the Heart Failure Association (HFA) of the ESC. Eur. Heart J..

[4] Johansson I., Joseph P., Balasubramanian K., McMurray J.J.V., Lund L.H., Ezekowitz J.A., Kamath D., Alhabib K., Genis A.B., Budaj A. (2021). Health-related quality of life and mortality in heart failure: The global congestive heart failure study of 23,000 patients from 40 countries. Circulation.

[5] Theofilis P., Oikonomou E., Tsioufis K., Tousoulis D. (2023). Diabetes Mellitus and Heart Failure: Epidemiology, Pathophysiologic Mechanisms, and the Role of SGLT2 Inhibitors. Life.

[6] Palazzuoli A., Evangelista I., Ruocco G., Lombardi C., Giovannini V., Nuti R., Ghio S., Ambrosio G. (2019). Early readmission for heart failure: An avoidable or ineluctable debacle?. Int. J. Cardiol..

[7] Khan S.S., Ning H., Shah S.J., Yancy C.W., Carnethon M., Berry J.D., Mentz R.J., O’Brien E., Correa A., Suthahar N. (2019). 10-Year Risk Equations for Incident Heart Failure in the General Population. J. Am. Coll. Cardiol..

[8] Uijl A., Koudstaal S., Direk K., Denaxas S., Groenwold R.H., Banerjee A., Hoes A.W., Hemingway H., Asselbergs F.W. (2019). Risk factors for incident heart failure in age-and sex-specific strata: A population-based cohort using linked electronic health records. Eur. J. Heart Fail..

[9] Magnussen C., Niiranen T.J., Ojeda F.M., Gianfagna F., Blankenberg S., Vartiainen E., Sans S., Pasterkamp G., Hughes M., Costanzo S. (2019). Sex-Specific Epidemiology of Heart Failure Risk and Mortality in Europe: Results from the BiomarCaRE Consortium. JACC Heart Fail..

[10] Yandrapalli S., Malik A.H., Namrata F., Pemmasani G., Bandyopadhyay D., Vallabhajosyula S., Aronow W.S., Frishman W.H., Jain D., Cooper H.A. (2022). Influence of diabetes mellitus interactions with cardiovascular risk factors on post-myocardial infarction heart failure hospitalizations. Int. J. Cardiol..

[11] Ohkuma T., Komorita Y., Peters S.A.E., Woodward M. (2019). Diabetes as a risk factor for heart failure in women and men: A systematic review and meta-analysis of 47 cohorts including 12 million individuals. Diabetologia.

[12] Chan J.S., Satti D.I., Lee Y.H., Hui J.M., Lee T.T., Chou O.H., Wai A.K., Ciobanu A., Liu Y., Liu T. (2022). High visit-to-visit cholesterol variability predicts heart failure and adverse cardiovascular events: A population-based cohort study. Eur. J. Prev. Cardiol..

[13] Liu H., Zhang J., Li Z., Liu J., Lian S., Le J. (2022). Association between remnant cholesterol and heart failure: A prospective cohort study. Front. Cardiovasc. Med..

[14] Castañer O., Pintó X., Subirana I., Amor A.J., Ros E., Hernáez Á., Martínez-González M.Á., Corella D., Salas-Salvadó J., Estruch R. (2020). Remnant Cholesterol, Not LDL Cholesterol, Is Associated with Incident Cardiovascular Disease. J. Am. Coll. Cardiol..

[15] Son Y.-J., Lee H.-J. (2020). Association between persistent smoking after a diagnosis of heart failure and adverse health outcomes: A systematic review and meta-analysis. Tob. Induc. Dis..

[16] Kao D.P., Lewsey J.D., Anand I., Massie B.M., Zile M.R., Carson P.E., McKelvie R.S., Komajda M., McMurray J.J., Lindenfeld J. (2015). Characterization of subgroups of heart failure patients with preserved ejection fraction with possible implications for prognosis and treatment response. Eur. J. Heart Fail..

[17] Shah S.J., Katz D.H., Selvaraj S., Burke M.A., Yancy C.W., Gheorghiade M., Bonow R.O., Huang C.C., Deo R.C. (2015). Phenomapping for novel classification of heart failure with preserved ejection fraction. Circulation.

[18] Peters A.E., Tromp J., Shah S.J., Lam C.S.P., Lewis G.D., Borlaug B.A., Sharma K., Pandey A., Sweitzer N.K., Kitzman D.W. (2023). Phenomapping in heart failure with preserved ejection fraction: Insights, limitations, and future directions. Cardiovasc. Res..

[19] Teramoto K., Teng T.H.K., Chandramouli C., Tromp J., Sakata Y., Lam C.S.P. (2022). Epidemiology and Clinical Features of Heart Failure with Preserved Ejection Fraction. Card. Fail. Rev..

[20] Uijl A., Savarese G., Vaartjes I., Dahlström U., Brugts J.J., Linssen G.C., Empel V., Rocca H.B., Asselbergs F.W., Lund L.H. (2021). Identification of distinct phenotypic clusters in heart failure with preserved ejection fraction. Eur. J. Heart Fail..

[21] Nouraei H., Rabkin S.W. (2021). A new approach to the clinical subclassification of heart failure with preserved ejection fraction. Int. J. Cardiol..

[22] Gomes I., Britto V. 2022 Census: Number of People Aged 65 or Over Grew 57.4% in 12 Years. Agência IBGE Notícias. https://tinyurl.com/4fybz224.

[23] Bocchi E.A., Brandão A.A., Mesquita E.T., Nakamuta J.S., Bichels A., Forestiero F.J. (2023). Heart Failure with Preserved Ejection Fraction in Brazil: A Systematic Review. ABC Heart Fail. Cardiomyop..

[24] Brasil Ministério da Saúde (2023). Secretaria de Vigilância em Saúde. Departamento de Análise em Saúde e Vigilância de Doenças Não Transmissíveis.

[25] Nogueira de Sá A.C., Gomes C.S., Moreira A.D., Velasquez-Melendez G., Malta D.C. (2022). Prevalence and factors associated with self-reported diagnosis of high cholesterol in the Brazilian adult population: National Health Survey 2019. Epidemiol. Serv.Saúde..

[26] D’Amato A., Prosperi S., Severino P., Myftari V., Labbro Francia A., Cestiè C., Pierucci N., Marek-Iannucci S., Mariani M.V., Germanò R. (2024). Current Approaches to Worsening Heart Failure: Pathophysiological and Molecular Insights. Int. J. Mol. Sci..

[27] Rohde L.E.P., Montera M.W., Bocchi E.A., Clausell N.O., Albuquerque D.C., Rassi S., Colafranceschi A.S., Freitas Júnior A.F., Ferraz A.S., Biolo A. (2018). Diretriz Brasileira de Insuficiência Cardíaca Crônica e Aguda. Arq. Bras. Cardiol..

[28] Cobas R., Rodacki M., Giacaglia L., Calliari L., Noronha R., Valerio C., Custódio J., Santos R., Zajdenverg L., Gabbay G. Diagnóstico do Diabetes e Rastreamento do Diabetes Tipo 2. https://diretriz.diabetes.org.br/diagnostico-e-rastreamento-do-diabetes-tipo-2/.

[29] Faludi A.A., Izar M., Saraiva J., Chacra A., Bianco H.T., Afiune A., Neto Bertolami A., Pereira A.C., Lottenberg A.M., Sposito A.C. (2017). Atualização da Diretriz Brasileira de Dislipidemias e Prevenção da Aterosclerose—2017. Arq. Bras. Cardiol..

[30] Barroso W.K., Rodrigues C.I., Bortolotto L.A., Mota-Gomes M.A., Brandão A.A., Feitosa A.D., Machado C.A., Poli-de-Figueiredo C.E., Amodeo C., Mion D. (2021). Diretrizes Brasileiras de Hipertensão Arterial–2020. Arq. Bras. Cardiol..

[31] Schuit A.J., van Loon A.J., Tijhuis M., Ocke M. (2002). Clustering of lifestyle risk factors in a general adult population. Prev. Med..

[32] Heinzel F.R., Shah S.J. (2022). The Future of Heart Failure with Preserved Ejection Fraction: Deep Phenotyping for Targeted Therapeutics. Herz.

[33] Meijs C., Handoko M.L., Savarese G., Vernooij R.W., Vaartjes I., Banerjee A., Koudstaal S., Brugts J.J., Asselbergs F.W., Uijl A. (2023). Discovering Distinct Phenotypical Clusters in Heart Failure Across the Ejection Fraction Spectrum: A Systematic Review. Curr. Heart Fail. Rep..

[34] Kou M., Wang X., Ma H., Li X., Heianza Y., Qi L. (2023). Degree of joint risk factor control and incident heart failure in hypertensive patients. JACC Heart Fail..

[35] Triposkiadis F., Xanthopoulos A., Parissis J., Butler J., Farmakis D. (2022). Pathogenesis of chronic heart failure: Cardiovascular aging, risk factors, comorbidities, and disease modifiers. Heart Fail. Rev..

[36] Nadruz W. (2015). Myocardial remodeling in hypertension. J. Hum. Hypertens..

[37] Schiffrin E.L. (2012). Vascular remodeling in hypertension. Hypertension.

[38] Aune D., Schlesinger S., Neuenschwander M., Feng T., Janszky I., Norat T., Riboli E. (2018). Diabetes mellitus, blood glucose and the risk of heart failure: A systematic review and meta-analysis of prospective studies. Nutr. Metab. Cardiovasc. Dis..

[39] Kenny H.C., Abel E.D. (2019). Heart Failure in Type 2 Diabetes Mellitus. Circ. Res..

[40] Aboumsallem J.P., Muthuramu I., Mishra M., De Geest B. (2019). Cholesterol-Lowering Gene Therapy Prevents Heart Failure with Preserved Ejection Fraction in Obese Type 2 Diabetic Mice. Int. J. Mol. Sci..

[41] Ribeiro G.J., Pinto A.A. (2021). Consumption of ultra-processed foods in Brazilian children: An analysis of regional trends. J. Pediatr Nurs..

